# Complicated Case of Obstetrics

**Published:** 1858-06

**Authors:** B. T. Smith

**Affiliations:** Central Institute, Ala.


### ARTICLE III.

*Complicated Case of Obstetrics.* By B. T. SMITH, M. D. Central Institute, Ala.

On the 6th Feb. last, I was called to see Mrs. ————, at whose residence I arrived about 7 o'clock, P. M. From her own statement I found she had been in labor about twelve or fifteen hours. As soon as I could make the preliminary arrangements, I proceeded to make an examination. I found the os uteri well dilated, the liquor amnii still entire. As I proceeded in my examination, I discovered the funis umbilicalis with its steady pulsations; and finally traced it to its origin in the fœtal surface of the placenta, which was then near the right ilium. At once I suspected it would be a case of placenta previa. In a few minutes I made another examination, that I might have a correct diagnosis of the case; and while this was going on, the waters broke and the funis descended. There was no time for hesitation. I saw, I thought, clearly, a case of prolapsus funis, and had labor been brisk, it could not have been otherwise, but fortunately, contractions were going on slowly. I made every effort for the recession of the cord, and in doing so, became satisfied of the proximity of the placenta. In this time the child's head had advanced, and the problem was, which would take precedence. One thing astonished me more than all—there was no hemorrhage.

Firmly fixing in my mind that I would give the advantage to the fœtus, I pressed the placenta, and wished strongly for an assistant, but it was a futile wish. I was nailed to the spot, and though the woman remonstrated—that I was suspending her "misery," yet I persisted, and finally a stronger pain came to my relief, and the fœtal head took possession of the superior strait in advance of the obtruder. As soon as I was satisfied of this, and found the presentation most favorable, I consoled the woman, and let nature take its course. In about forty minutes the cries of a new-born child were heard. No difficulty was encountered in the delivery, except relieving the neck from the envelopes of the cord. At about half-past eleven, the child was given to the nurse. I suffered about twenty minutes to elapse before I attempted to take away the placenta. Making gentle traction on the cord, I brought it out its full length, and came to its insertion in the placenta, which was still within the os uteri. I felt now that there was to be some delay, and I was not disappointed. I waited nearly two hours, making frequent efforts by traction on the one hand, and using means to contract the uterus on the other, but all was abortive. I introduced my hand and found the uterus pretty closely drawn upon the placenta; I grasped the body, and expected to have my hand and it thrust out together. But judge of my disappointment—it refused to come, or even yield. I could have torn it to pieces, but I did not wish to do this. It was firmly attached to the cervix, by what seemed to me a cartilaginous adhesion. Feeling round it gave no pain, but every attempt to break down the adhesion produced intense suffering. At length, by the entreaties of the woman, and my own weariness, having worked seven hours after delivery, I concluded to let a little time elapse. I reclined upon a bed to rest, and perhaps an hour elapsed before I resumed my efforts. And now I found a new difficulty; I could not introduce my hand on account of the contraction of the parts. I applied a ligature, and severed the cord, leaving enough to answer my purposes. I scarcely knew what to do for the best. I gave liberal doses of ergot et bi. borate of soda, if so be, by strong efforts of the uterus, the adhesions might be overcome. In four hours, powerful efforts were produced, and in one of those powerful throes, amounting almost to convulsion, the adhesions were overcome, and the placenta was cast out. And strange to tell, little more hemorrhage than usual in simple cases. I gave a composing draught, and left. The officiousness of the nurse prevented me from examining the nature of the adhesions critically; excepting that two days after there was a suppression of the lochia, which was soon re-established, nothing serious resulted, and the woman speedily recovered.

